# Biosensors for European Zoonotic Agents: A Current Portuguese Perspective

**DOI:** 10.3390/s21134547

**Published:** 2021-07-02

**Authors:** Samuel da Costa Miguéis, Ana P. M. Tavares, Gabriela V. Martins, Manuela F. Frasco, Maria Goreti Ferreira Sales

**Affiliations:** 1 BioMark@ISEP, School of Engineering, Polytechnic Institute of Porto, 4249-015 Porto, Portugal; uc45757@uc.pt (A.P.M.T.); gfdvm@isep.ipp.pt (G.V.M.); mffrasco@uc.pt (M.F.F.); 2 Centro de Investigação Desenvolvimento e Inovação da Academia Militar, Academia Militar, Instituto Universitário Militar, 1169-203 Lisboa, Portugal; 3 BioMark@UC, Faculty of Sciences and Technology, University of Coimbra, 3030-790 Coimbra, Portugal; 4 CEB, Centre of Biological Engineering, University of Minho, 4710-057 Braga, Portugal

**Keywords:** biosensors, zoonoses, Portugal, pathogenic bacteria, real-time multiple detections

## Abstract

Emerging and recurrent outbreaks caused by zoonotic agents pose a public health risk. They result in morbidity and mortality in humans and significant losses in the livestock and food industries. This highlights the need for rapid surveillance methods. Despite the high reliability of conventional pathogen detection methods, they have high detection limits and are time-consuming and not suitable for on-site analysis. Furthermore, the unpredictable spread of zoonotic infections due to a complex combination of risk factors urges the development of innovative technologies to overcome current limitations in early warning and detection. Biosensing, in particular, is highlighted here, as it offers rapid and cost-effective devices for use at the site of infection while increasing the sensitivity of detection. Portuguese research in biosensors for zoonotic pathogens is the focus of this review. This branch of research produces exciting and innovative devices for the study of the most widespread pathogenic bacteria. The studies presented here relate to the different classes of pathogens whose characteristics and routes of infection are also described. Many advances have been made in recent years, and Portuguese research teams have increased publications in this field. However, biosensing still needs to be extended to other pathogens, including potentially pandemic viruses. In addition, the use of biosensors as part of routine diagnostics in hospitals for humans, in animal infections for veterinary medicine, and food control has not yet been achieved. Therefore, a convergence of Portuguese efforts with global studies on biosensors to control emerging zoonotic diseases is foreseen for the future.

## 1. Introduction

Man has been in search of security and a healthy life to ensure the survival of the species. Over the centuries, various tools have been developed to achieve this goal, and biosensor research has taken a leading role in measuring critical parameters to improve the well-being of modern societies. The emergence of the first sensor dates back to the 17th century with the thermometer’s invention [1]. Since then, there has been an exponential development in analytical chemistry and sensing devices. Consequently, the appearance of new sensors was inevitable, and the production of the first “true” biosensor by Leland C. Clark, Jr. occurred in 1956 for oxygen detection, consolidated by the pioneering work of Clark and Lyons, published in 1962, which described an amperometric enzyme electrode for the detection of glucose [2]. Nowadays, biosensors are defined as “analytical devices incorporating a biological material, a biologically derived material or a biomimic as a recognition molecules, which is either intimately associated with or integrated within a physicochemical transducer or transducing microsystems” [3].

Biosensors have many useful applications, including detecting bacteria and viruses for various purposes, such as disease prevention and diagnostics, food pathogen identification, food safety, and hygiene control. They can also be used in theranostics, a combination of detection and delivery of therapeutics, which involves monitoring infected wounds and delivering drugs as needed [1,4]. Concerning the food industry, biosensors can be extremely helpful by preventing food fraud (e.g., adulteration, substitution, dilution) and contamination. For example, rapid, inexpensive, and portable devices that alert in response to contamination and immediately stop the distribution or sales process can effectively protect consumers from health complications and the industry from economic loss [5]. The use of biosensors to prevent labelling errors in fish species illustrates their usefulness in protecting against food fraud, as such errors can reach levels in the range of 70% in the European Economic Area [6], 40% in Ireland, and 11% in the United Kingdom [7], 20% in Canada [8], and 6% in the European Union (EU) [9]. According to the World Health Organization (WHO), nearly 1 in 10 people contracted a foodborne illness in 2010 due to 31 different foodborne hazards: viruses, bacteria, parasites, chemicals, and toxins. The most commonly reported pathogens are norovirus and *Campylobacter* spp. Non-typhoidal *Salmonella* is the deadliest foodborne pathogen [10].

**Figure 1:**
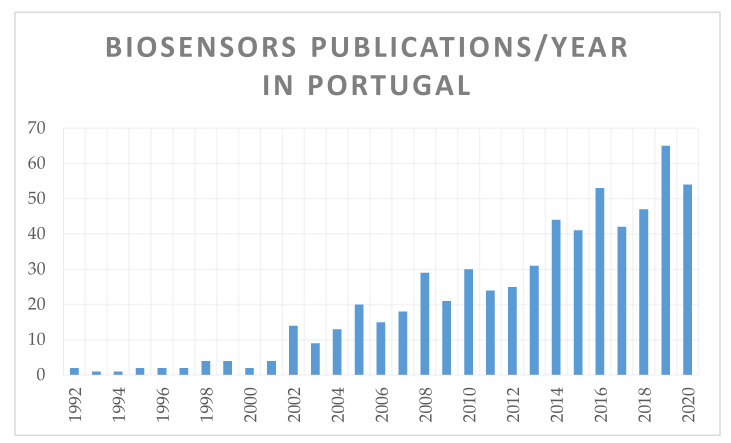
Data obtained from webofknowledge.com from 630 records cross-searching keywords: ALL FIELDS: (biosensor) and ADDRESS: (Portugal) in Web of Science [v.5.34].

The threat of zoonotic pathogens to global health requires the development of sensitive, selective, and portable biosensors. To date, various approaches have been developed worldwide for the identification and quantification of bacteria and viruses. Portuguese research in biosensors has been on the rise since 1992 (Figure 1). Within the topic of biosensors, Portuguese publications represent only ~1% of the worldwide publications, focusing on analytical chemistry and electrochemistry (Table 1). Electrochemical and optical biosensors are among the most studied biosensors due to their sensitive and selective detection properties, low cost, portability, and high accessibility, allowing them to be used by untrained individuals without expensive technology [11,12].

Therefore, this review provides an overview of the most commonly reported pathogenic bacteria, their occurrence in the EU, and the negative consequences of their widespread contamination and infection. At the same time, the biosensors developed by Portuguese teams for the detection of these bacteria are reviewed and discussed, taking into account future developments in this field.

## 2. Sensors for Commonly Reported Pathogenic Bacteria

The most recent 2021 zoonoses report by the European Food Safety Authority (EFSA) presented *Salmonella* Enteritidis and *Campylobacter jejuni* as the most commonly reported bacteria zoonosis, followed by Shiga-toxin-producing *Escherichia coli*, *Listeria monocytogenes*, and *Yersinia enterocolitica*, and with a lower frequency of bovine tuberculosis (*Mycobacterium* spp.), *Brucella*, *Coxiella burnetii* (Q fever), *Chlamydia* spp., *Clostridium* spp., *Enterococcus*, *Erysipelothrix*, *Proteus*, *Staphylococcus* spp., Tularaemia (*Francisella tularensis*), and *Cronobacter sakasakii*. Regarding viral zoonoses in the EU, EFSA identifies rabies and West Nile Virus as the most prevalent pathogens, except for the recent pandemic caused by SARS-CoV-2, with a lower prevalence of tick-borne encephalitis virus [13].

**Figure 2:**
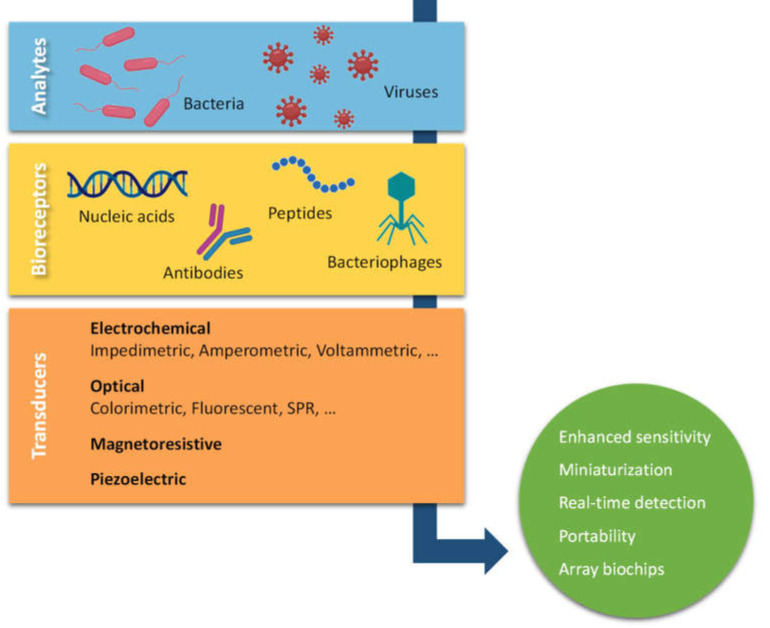
Schematic representation of common biosensors for improved detection of zoonotic pathogens.

Rapid, sensitive, and selective biosensors for detecting pathogenic bacteria and viruses are essential to reduce their impact on human health and improve the effectiveness of prevention and treatment, especially considering how useful they are in point-of-use testing (Figure 2). The biological differences between so many types of zoonotic pathogens are a challenge for sensor fabrication. Nevertheless, exciting devices have been developed and are discussed in the following sections.

### 2.1. Salmonella

Two species of *Salmonella* are recognised as pathogenic: *Salmonella bongori* and *Salmonella enterica*. *Salmonella enterica* can be divided into seven subspecies according to biotype (I, II, IIa, IIIb, IV, VI, and VII), with subspecies I (*Salmonella choleraesuis* subsp. *choleraesuis*, or widely accepted nomenclature *Salmonella enterica* subsp. *enterica*) responsible for 99% of all human salmonellosis. Strains of subspecies I colonise a wide variety of hosts. Serovar Typhimurium infects many mammalian species, but serovar Typhi infects only humans [14]. These rod-shaped Gram-negative bacteria have an optimal growth temperature range between 35 and 40 °C. In some food matrices, it has been described that some strains can grow between 2 and 54 °C [15]. However, there is no consensus, as the absence of growth in foods stored at 4.6 °C has also been reported [16]. 

EFSA reported that, in 2019, in the 28 European member states, the main serovars responsible for salmonellosis in humans were *Salmonella* Enteritidis and *Salmonella* Typhimurium, which account for 50.3% and 11.9% of cases, respectively [13]. Salmonellosis remains the second most common zoonosis in humans in the EU. Eggs and egg products are the most common carriers of salmonellosis, accounting for 37% of foodborne outbreaks (FBO) [13]. FBOs associated with *Salmonella* in infant food continue to occur in the EU, with lower percentage impact but high importance, as shown by the transnational outbreak of *Salmonella* Poona in 2019 [13].

An electrochemical immunosensor combining magnetic nanoparticles (MNPs) and CdS labels was developed for the detection of *Salmonella* Typhimurium, and achieved a detection limit (LOD) of 13 cells/mL [17]. In this work, the detection of *Salmonella* was performed in two steps of the binding process between bacteria and the bioreceptor. First, specific antibodies against bacteria were bound to iron/gold core/shell (Fe@Au) nanoparticles and CdS nanocrystals. Due to the magnetic properties of the Fe@Au nanoparticles, the application of an external magnetic field allowed rapid separation and subsequent detection of the bacteria. The CdS nanocrystals were used as markers for signal amplification by square-wave anodic stripping voltammetry (SWASV). This labelled electrochemical method allowed rapid and sensitive determination of *Salmonella* Typhimurium in spiked milk samples [17]. 

An exciting aspect made possible by developing a phage-based device is the ability to detect viable *Salmonella* Enteritidis cells in dormant states, i.e., viable but nonculturable (VBNC) [18]. In this work, bacteriophages were used as bioreceptors to distinguish viable and VBNC cells from dead cells in a magnetoresistive (MR) biochip. The bacteriophages were first immobilised on gold surfaces functionalised with sulpho-LC-SPDP (sulphosuccinimidyl 6-[3′-(2-pyridyldithio)-propionamido] hexanoate) and showed the ability to recognise viable plus VBNC cells. In addition, *Salmonella* was also detected using a sandwich assay, in which cells were first incubated with immobilised phages on an MR chip, and detection was based on antibodies labelled with MNPs. As a result of biorecognition, the magnetic fringe field generated by the labels was detected as a change in resistance of the sensor. This sensor allowed the determination of up to 3–4 cells/sensor with less sample volume than the traditional flow cytometry method [18]. 

Despite improvements in separation and rapid detection methods for *Salmonella*, the most advanced methods still lack the necessary properties to meet regulations for food analysis applications. Among these methods, electrochemical biosensors have been mentioned as a promising reliable technology for further validation and commercialisation, especially considering the low LODs achieved and the possible incorporation of different bioreceptors (e.g., antibodies, aptamers, bacteriophages) [19]. The high sensitivity, automation, and real-time detection in complex samples are the main challenges faced by each of the sensors under consideration before implementation [20].

### 2.2. Escherichia coli

*Escherichia coli* is a versatile organism that can exist as an aerobe, anaerobe, or facultative anaerobe, and ferments sugars and amino acids [21]. It is a mesophilic organism that multiplies at temperatures ranging from 7 to 45 °C. Some serotypes are responsible for three types of human diseases: (i) neonatal meningitis, (ii) chronic urinary tract infections, and (iii) gastroenteritis [22,23]. The pathogenic *E. coli* associated with human gastroenteritis are classified into six classes: (i) enteropathogenic *E. coli* (EPEC), (ii) enteroaggregative *E. coli* (EAEC), (iii) enteroinvasive *E. coli* (EIEC), (iv) enterotoxinogenic *E. coli* (ETEC), (v) enterohemorrhagic *E. coli* (EHEC), and (vi) diffusely adherent *E. coli* (DAEC) [24,25,26,27,28]. Outbreaks of EHEC (O157:H7) have been frequently reported in industrialised countries. Other strains, such as Shiga toxin producers, called Shiga-toxin-producing *E. coli* (STEC) or verocytotoxin-producing *E. coli* (VTEC), such as *E. coli* O104:H4, have also caused outbreaks, but less frequently. In the recent EFSA report, STEC infection is ranked third [13]. In addition to their application in the context of food safety, potential biosensors for *E. coli* could also represent a significant advance for disease prevention in companion animals and animal production. For example, untrained personnel could use biosensors at the point-of-use to prevent or detect mastitis in cattle [29].

**Figure 3:**
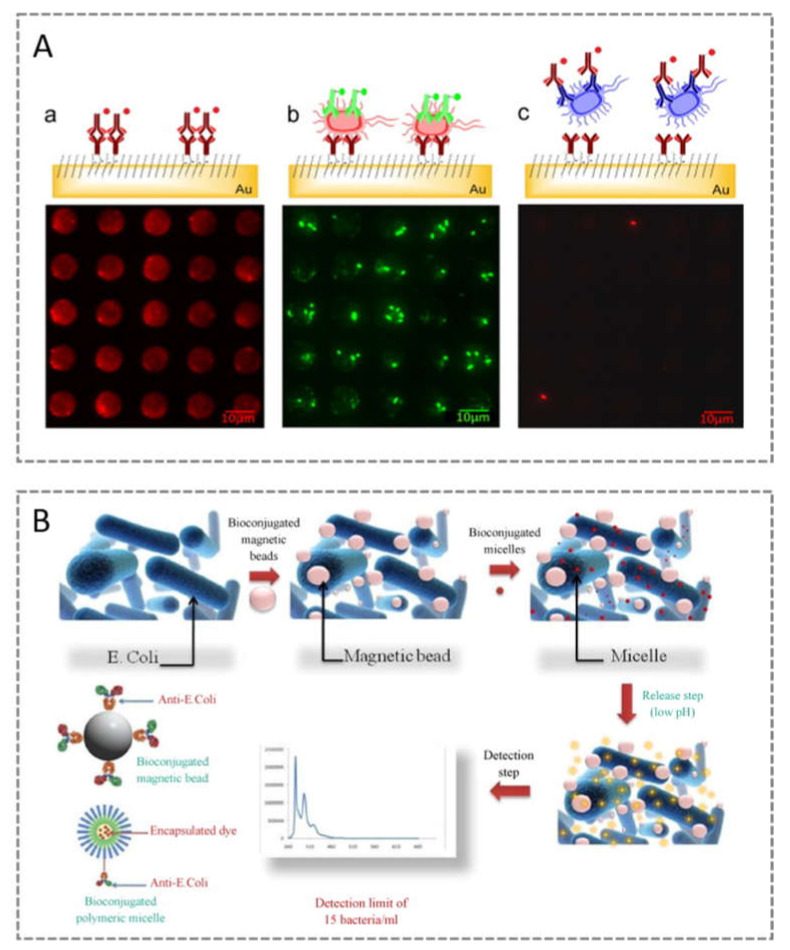
Immunoassays for detection of *E. coli*: (**A**) fluorescence microscopy images of anti-*E. coli* antibodies patterned on gold surfaces and probed with anti-rabbit (a); patterned anti-*E. coli* antibodies selectively recognise *E. coli* O157:H7 labelled with a secondary FITC-conjugated antibody (b); and the array presented low grafting capacity towards a non-specific bacteria such as *Salmonella* Typhimurium (c) (reproduced with permission from Barreiro dos Santos et al. (2013) [30], Copyright 2013, Elsevier B.V.); (**B**) Schematic diagram of a new bioassay for capturing target bacteria, which relied on using target-specific, dye-loaded polymeric micelles and magnetic beads with a capturing reagent (reproduced with permission from Mouffouk et al. (2011) [36], Copyright 2011, Elsevier B.V.).

Detection of microorganisms using antibodies is a relatively successful and widely used technology to construct extremely sensitive biosensors. During their assembly, the procedure for immobilising the antibodies on the transducer platform plays a crucial role in the analytical performance of the sensing device. Barreiro dos Santos et al. (2015) developed a label-free immunosensor for the detection of *E. coli* O157:H7 by performing a covalent immobilisation of the antibody directly on the gold surface electrode through a self-assembling monolayer (SAM) of mercaptohexadecanoic acid [30]. Subsequently, the electrochemical impedance spectroscopy (EIS) technique enabled the quantification of *E. coli* O157:H7 bacteria with an extremely low LOD of 2 CFU/mL [30] (Figure 3A).

Another electrochemical label-free detection of *E. coli* bacteria was investigated using impact electrochemistry [31]. In this study, *N*,*N*,*N*′,*N*′-tetramethyl-para-phenylenediamine (TMPD) was used as a redox mediator. It interacts with bacterial cytochrome c oxidases, resulting in electrochemical current “on” signals in the presence of *E. coli*. This approach has the major advantage of minimising false positive signals due to non-electroactive impurities, and eliminates the step of cell lysis. Further developments of this work were presented by Kuss et al. (2019), who developed a rapid, selective, and inexpensive detection method for pathogenic bacteria expressing cytochrome c oxidase by combining immunohistochemistry and electrochemical sensing. With the possibility of a point-of-use application in mind, the team developed a biosensor with screen-printed electrodes (SPEs), promoting a low-cost and portable technology. Antibodies to *E. coli* were immobilised on gold-based SPEs using thiol chemistry, and a significant increase in electrochemical current was measured after binding the bacteria. This technique could allow the detection of all bacteria that exhibit cytochrome c oxidase, as was the case with *Neisseria gonorrhoeae*, thus enabling its use as the first warning agent for potential contamination events [32].

As an alternative approach to antibodies, Queirós et al. (2013) used aptamers as recognition material for the construction of impedimetric sensors [33]. In this case, two DNA aptamer sequences, designated ECA I and II, were tested for the detection of *E. coli* outer membrane proteins (EcOMPs). A gold electrode platform was used to perform immobilisation of the aptamer capture probe. Then, the performance of each aptamer probe was evaluated by tracking the variation of electron transfer behaviour before and after the binding of EcOMPs. In addition to their selectivity and regeneration properties, this label-free biosensor enabled a linear response range of 1 × 10^−7^–2 × 10^−6^ M, which shows great promise for the determination of EcOMPs in water samples. 

Another interesting approach concerns the preparation of impedimetric immunosensors based on indium tin oxide (ITO) surfaces for the detection of pathogenic bacteria. Barreiro dos Santos et al. (2013) described a label-free immunosensor for the detection of low levels of *E. coli* O157:H7 on ITO electrodes. Anti-*E. coli* antibodies were immobilised on ITO electrodes after the covalent binding of epoxysilane on the ITO surface [30]. The detection capacity of the ITO-based immunosensor was evaluated by EIS and revealed a linear response range of 10–10^6^ CFU/mL, with an LOD of 1 CFU/mL [34].

In parallel, optical biosensors have undergone significant developments to meet the need for compact, low-cost devices that can be easily operated on the bench. In this context, a paper reported the direct detection of genomic DNA (gDNA) from *E. coli* without amplification steps. This approach was achieved by combining polymerase activity and solid-phase DNA hybridisation. The gDNA from *E. coli* was first hybridised to surface-immobilised complementary probes, followed by template-mediated extension at the 3′ terminus of the probe due to Klenow I polymerase activity specificity. Initially, colourimetric detection was pursued, but, due to the semi-quantitative result, this was replaced by a fluorescent detection scheme that allowed a detection limit of 5 pM gDNA [35]. Another interesting fluorescent biosensor was developed by Mouffouk et al. (2011), using self-assembled pH-responsive polymeric micelles and MNPs, both surface-modified with anti-E.coli antibodies [36]. The preparation of poly(ethylene glycol-b-trimethylsilyl methacrylate) micelles allowed the encapsulation of a significant amount of hydrophobic fluorescent tracers. The bioconjugated magnetic beads were first used to capture the bacteria, and then mixed with the antibody-labelled micelles. After washing the unbound micelles, the release of the encapsulated dye and fluorescence measurements were achieved by lowering the pH to 5.0 (Figure 3B). This approach offers a great advantage in terms of sensitivity performance due to the amplification effect produced by the optical signal from millions of fluorophores, and allows for an LOD of 15 bacteria/mL [36].

In another work, the application of a fibre optic sensor with evanescent waves for the detection of EcOMP was demonstrated by Queirós et al. (2014) [37]. A refractometric platform was functionalised with an *E. coli* DNA aptamer (ECA) using two different methods of immobilisation: (i) electrostatic assembly with a cationic polymer, and (ii) covalent binding using an organofunctional alkoxysilane molecule. Subsequently, self-assembly of the ECA and consecutive binding of the EcOMP to the bare refractometric surface increase the effective refractive mode of the cladding, resulting in a shift in resonance wavelength as a function of the increase in surface mass. This label-free biosensor enabled the detection of EcOMP in water over the concentration range of 0.1 nmol/L to 10 nmol/L [37].

The development of microfluidic biosensing platforms has received a significant boost as they provide a quick and efficient alternative to the culture methods used to identify different types of pathogens. Moreover, the integration of these microfluidic systems with magnetic labelling has enabled the production of a versatile sensor for the detection of *Enterobacteriaceae* [38]. In this study, bacteria were labelled with magnetic microparticles functionalised with antibodies targeting surface antigens of *E. coli*, which allowed specific capture. The complex was then injected into a microfluidic channel and set in motion by applying a magnetic field. The change in velocity of the microparticles was correlated with the loading of the respective pathogenic bioanalyte [38]. 

However, integrated optical systems based on interferometers may also be a promising approach in the optical field to detect specific pathogens, such as *E. coli*. Recently, Bastos et al. (2018) fabricated an optical sensor based on a Mach–Zehnder interferometer by printing the device pattern on transparent, self-patternable organic–inorganic di-ureasil hybrid films using a direct UV laser [39]. The main advantages of this approach include the low cost of the technology. As an alternative to more conventional lithographic techniques, this novel approach offers reduced process complexity, mild conditions, and no need for expensive equipment. The performance of the sensor was evaluated using the growth of *E. coli* cells in an aqueous medium, where the measured sensitivity (2 × 10^−4^ RIU) and LOD (2.0 × 10^3^ cells/mL) are among the known values for low refractive index contrast integrated optics-based solutions [39].

### 2.3. Listeria Monocytogenes

*Listeria* spp. is an anaerobic, microaerophilic, and facultative anaerobic Gram-positive, non-spore-forming rod bacterium, consisting of six species: *L. monocytogenes*, *L. ivanovii*, *L. innocua*, *L. welshimerri*, *L. seeligeri*, *L. grayi*, and *L. monocytogenes*. Another key property of these ubiquitous microorganisms is their ability to grow at freezing temperatures. *Listeria monocytogenes sensu stricto* belongs to genomic group 1 [40] and includes strains of serovars 1/2a, 1/2b, 1/2c, 3a, 3b, 3c, 4a, 4ab, 4c, 4d, 4e, and 7 [41].

Epidemiological studies have shown that listeriosis in humans can be a foodborne illness [42,43], and infection usually occurs through the consumption of contaminated ready-to-eat (RTE) foods [44]. There are many points of contamination in the RTE food chain. Still, the food processing environment appears to be of particular importance as a source of *L. monocytogenes* and their introduction into the food system [45].

Strains of serotype 4b have been reported as the primary cause of 60% of human listeriosis [46,47]. Outbreaks have been associated with contaminated coleslaw [48], soft cheeses [49,50,51], pâté [52], and pork tongue [53]. Reporting listeriosis is mandatory in the EU, and there is evidence of an increased number of foodborne outbreaks in recent years (e.g., 21 in 2019, 14 in 2018, and 10 in 2017). The food vehicles for these outbreaks in European member states are “meat and meat products”, “broiler meat and products thereof”, “bovine meat and products thereof”, “pig meat and products thereof”, “mixed food products”, and “vegetables and juices and other products thereof” [13].

Listeriosis caused by strains of serotype 1/2b is not as common, representing 17% of cases [46], and is often associated with contaminated milk [54], rice salad [55], and imitation crab meat [56]. Listeriosis remains the foodborne illness in Europe with the highest case fatality and hospitalisation rates, especially in the age group over 64 years. The number of deaths increased by 31% in 2019 (300 deaths) compared to 2018 (229 deaths) [13].

Biosensors could be the solution to tackle such increased mortality rates, especially if it is possible to introduce a simple test on the food package or a cheap and quick point-of-use food test. The gold standard method takes 48–72 h to provide results, which is not compatible with the short shelf life of fresh products. The industry selling meat, meat products, and fish, which are the primary source of infections in the EU [13], needs to look for alternatives to avoid losing days of shelf life in their facilities until results are available. Among the currently available biosensor-based methods for *Listeria* identification, optical sensors offer better sensitivity despite their cost and performance [57].

As far as we know, the only Portuguese work aimed at detecting *L. monocytogenes* bacteria in milk samples was published in 2020 [58]. An electrochemical immunosensor was proposed in this work, aiming to quantify the invasion-associated protein p60 secreted by this pathogen. For this purpose, monoclonal and polyclonal antibodies specific for p60 proteins of *L. monocytogenes* and *Listeria* spp., respectively, were immobilised on SPEs. An additional secondary antibody conjugated to the enzyme reporter (alkaline phosphatase) was used to detect the presence of 3-indoxyl phosphate/silver ions. Under the optimised conditions, the immunosensor showed a linear response over the concentration range from 5 to 150 ng/mL, which allowed an LOD of 1.5 ng/mL. Finally, the electrochemical device was successfully applied to the analysis of spiked commercial milk samples, demonstrating their potential for rapid detection of *L. monocytogenes* in routine food quality control [58].

### 2.4. Staphylococcus spp.

Members of the genus *Staphylococcus* belong to the family *Micrococcaceae* and are Gram-positive, catalase-positive, and immotile cocci that occur singly, in pairs, or as irregular clusters. The optimal growth temperature is 37 °C, with a range of 6 to 48 °C, although reports suggest that this microorganism can only grow below 10 °C under certain conditions [59]. Nevertheless, enterotoxin production occurs up to 10 °C [60]. The genus *Staphylococcus* currently includes 36 recognised species and subspecies [61]. Staphylococcal food intoxication is caused by one or more *Staphylococcus Enterotoxin* (SE). There are 13 new SE and related proteins (types G through R and U), three new variants of SEC (SEC-bovine, SEC-ovine, and SEC-caprine), and four additional toxins (types Gv, Iv, Nv, and Uv) [59]. *Staphylococcus aureus* is ubiquitous, and although it is mainly found in primates, specific ecovars or biotypes can occasionally be found in various domestic animals or birds [62]. *S. aureus* has a niche preference for anterior nostrils in humans, especially in adults, and the skin [63,64]. In Europe, *S. aureus* intoxication was responsible for 1.4% of all outbreaks and is seventh in the top 10 pathogen/food pairings that caused the most hospitalisations in 2019 [13]. The trend of antibiotic resistance in the EU is decreasing for both species *S. aureus* and *S. pneumoniae*. However, methicillin-resistant *Staphylococcus aureus* (MRSA) is still present in 15% of total isolates, and penicillin non-wild-type, macrolide, and combined resistance in *S. pneumoniae* are 12.1%, 14.5%, and 7.2%, respectively. Despite this positive trend, MRSA still presents high morbidity and mortality rates, mainly due to bloodstream infections. In particular, in Portugal, MRSA isolates are higher than the EU median, above 25% [65]. 

**Figure 4:**
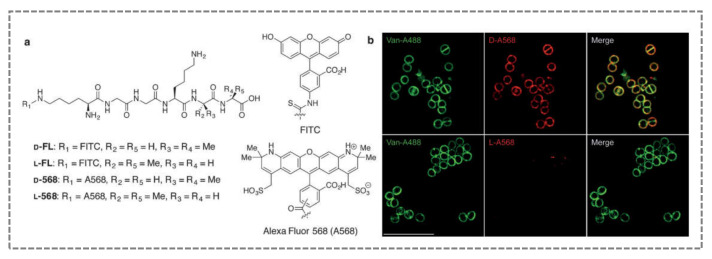
Labelled cell wall of *S. aureus* with stereoselective fluorescent stem peptide mimics: (**a**) chemical structures of probes D-FL and L-FL; D-A568 and L-A568; (**b**) representative images of structured illumination microscopy of *S. aureus* Newman labelled for 1 h with D-A568 (top panels) or L-A568 (bottom panels). Both samples were treated with Van-A488, which stains the entire cell wall of S. aureus (left panels). Scale bar: 5 µm (reproduced with permission from Gautam et al. (2015) [66], Copyright 2015, Wiley-VCH).

Gautam et al. (2015) used pulse labelling to investigate the spatial and temporal localisation of penicillin-binding protein 4 (PBP4) activity in living cells, providing new insights into the bacterial cell wall [66]. These authors developed an activity-based probe responsible for incorporating fluorophores at precise peptidoglycan (PG) crosslinking sites. Crosslinking of PG is catalysed by penicillin-binding proteins (PBPs) and is an important target of antibiotics, because crosslinks are essential for bacterial growth and survival. Fluorescent stem peptide mimics (FSPMs) of the natural substrate of PBPs were successfully incorporated into the cell wall of *S. aureus* (Figure 4). Interestingly, the incorporation of FSPMs provided stereoselective and specific signals for a single PBP, PBP4. 

**Figure 5:**
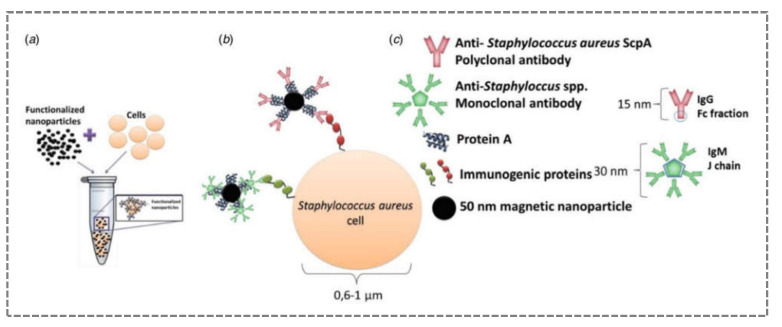
Scheme representing the immunomagnetic detection of cells: functionalised MNPs are incubated with bacterial cells (**a**), biological affinities between MNPs functionalised with different antibodies and bacterial cell wall immunogenic proteins (**b**), and predictable protein A binding site to each antibody (**c**) (reproduced with permission from Duarte et al. (2017) [67], Copyright 2017, Cambridge University Press).

From a deeper industrial perspective, the dairy products industry is eager to use biosensors in real-time analysis to detect *S. aureus* infections in the early stages and prevent the massive use of antibiotics. Livestock production can be severely affected by the presence of *S. aureus*, as shown by the economic losses due to mastitis in cattle. In 2016, an immunosensor based on magnetic detection of *S. aureus* in raw milk samples was developed and validated [67]. The approach adopted was based on immunomagnetic detection (Figure 5). MNPs were functionalised with specific antibodies. The analysis was performed using a lab-on-a-chip MR cytometer with microfluidic sample handling, which has been shown to detect the presence of bacteria above 100 CFU/mL [67].

### 2.5. Mycobacterium spp.

The genus *Mycobacterium* has nearly 150 species, which can be divided into fast-growing organisms and slow-growing organisms. The latter includes most species associated with human tuberculosis: *M. avium*, *M. intracellulare*, *M. leprae*, *M. marinum*, *M. ulcerans*, *M. kansai*, and organisms of the *M. tuberculosis* complex (MTC). The former includes only *M. abcessus* as an important human pathogen [68]. Species of MTC include *M. tuberculosis*, *M. africanum*, *M. bovis*, *M. microti*, *M. canettii*, *M. caprae*, *M. pinnipedii*, *M. suricattae*, *M. mungi*, *M. dassie*, and *M. oryx* [69]. In 2019, tuberculosis (TB), a disease caused by MTC, killed nearly 1.2 million people among HIV-negative people [70], and epidemiological data estimate that almost a quarter of the world population has latent TB infection [71], of whom only less than 10% progress to active TB during their lifetime. In addition, WHO reports that the pandemic due to SARS-CoV-2 could increase deaths due to the disruption of health services [70].

In Portugal, actions to reduce the numbers of TB still need to be improved, as the incidence in 2019 was still over 10 per 100,000 [70], which is higher than in most European countries. The most commonly used test to identify TB is direct Ziehl–Neelsen (ZN) sputum smear microscopy, with an LOD of 10^4^–10^5^ cells/mL [72,73]. Although ZN is the most commonly used diagnostic test, a challenge remains in patients with false-negative sputum results, such as HIV patients, extrapulmonary TB, and children. These require a different diagnostic approach, such as a rapid molecular test [70]. In addition, the ZN protocol requires an elaborate technique, carries the risk of human identification errors, and the risk of infection. These limitations have driven the search for alternative approaches that are faster, less expensive, and more sensitive. 

**Figure 6:**
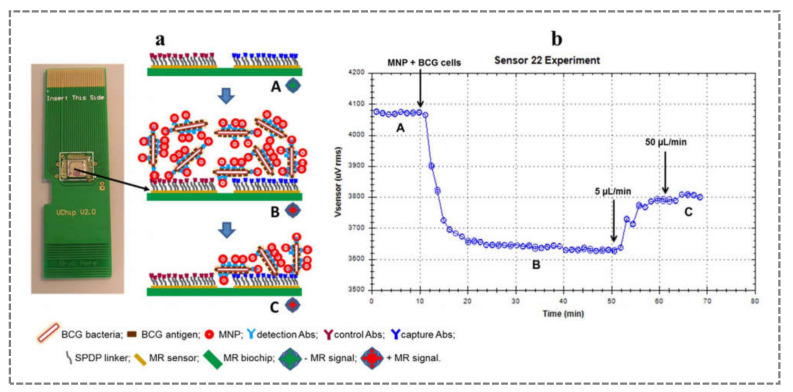
Image of an MR-biochip with the scheme of the sandwich-immunoassay reactions (**a**), and the corresponding average voltage variation curves over time, in the right area of the sensors whose surface was functionalised with capture antibodies (**b**): step A is the baseline or the negative MR signal (no label); then, the voltage drop is observed in step B due to positive MR signal (MNP@Abs@BCG in contact with the MR-biochip surface); and in step C, the non-bounded MNP are washed out and only the positive MR signal from labelled targets is recorded (MNP@Abs@BCG bonded to specific Abs on the biochip surface) (reproduced with permission from Barroso et al. (2018) [72], Copyright 2018, Elsevier B.V.).

Biosensors meet the necessary criteria to improve TB diagnosis, and Portuguese researchers have developed several biosensors for this purpose. MR biochips were developed in analogy with an enzyme-linked immunosorbent assay (ELISA), but with promising features, such as high sensitivity, miniaturisation, and portability [74]. In MR biochips, the enzymatic labelling of the ELISA assay is replaced by MNPs, and the sensor design can follow similar sandwich immuno-based formats. The general principle of operation is based on immobilising specific capture antibodies (anti-*M. tuberculosis*) on MNPs by streptavidin–biotin affinity interaction. This type of immobilisation provides a significant advantage in the detection of the target molecule. It allows the orientation of the antibody, which increases the availability of binding sites of the antibody on the material surface, and it improves the performance of the biosensor with respect to LOD (Figure 6). The recognition process begins after the MNPs with anti-*M. tuberculosis* capture the target bacteria. Then, the resulting immune complex is recognised by a secondary antibody previously immobilised on the surface of the MR biochip platform. An array of spin-valve sensors is responsible for detecting magnetically labelled cells by changes in the magnetic field. This MR biochip can achieve an estimated LOD below 10^4^ cells/mL, similar to the thresholds achieved when performing the standard ZN sputum smear microscopy assay, so future translation into the clinic is contemplated [72]. 

An optical detection method for *M. tuberculosis* was investigated by detecting a specific DNA sequence. Bernacka-Wojcik et al. (2010) integrated a dye-sensitised TiO_2_ photodetector with a Au-nanoprobe (DNA-functionalised Au nanoparticles) based on the non-crosslinking hybridisation method [75]. In this study, the fabrication of the photodetectors was followed by the traditional “doctor-blade” method and inkjet printing. The biorecognition element consisted of a specific DNA sequence complementary to the *M. tuberculosis* target DNA covalently bound to Au nanoparticles, resulting in the Au-nanoprobe. The Au nanoprobes aggregate in a solution with increased ionic strength, resulting in a visible colour transition from red to blue. In the presence of the complementary target DNA, this colour change is prevented, and the solution remains red [76,77]. In the mounted colourimetric sensors, both photodetectors showed sensitivity limits comparable to conventional spectrophotometric methods. Nevertheless, concentrations down to 1.0 nmol/L were detected with the “doctor-blade” system, while a limit of 1.5 nmol/L was reached with inkjet printing. Thus, the studied platform represents a technology with significant cost and time savings for DNA detection [75]. The relevance of using portable DNA sensing devices was achieved in a low-cost optoelectronic platform [78] and a lab-on-chip device [75]. These devices incorporated the operating principle of the biorecognition process in the form of Au-nanoprobes for colourimetric MTC DNA analysis. The optoelectronic platform consisted of an amorphous/nanocrystalline silicon photodetector integrated with a dual-colour-tuned RGBA LED as the light source, and the remaining components for signal acquisition and full automation. The prototype demonstrated that quantitative analysis down to 50 nmol/L is possible [78]. With the bio-microfluidic platform fabricated using replica moulding technology, the colourimetric DNA assay was miniaturised, requiring only 3 µL of DNA solution at a concentration of 30 ng/µL [75].

In another approach, Prabowo et al. (2021) reported a graphene-based portable surface plasmon resonance (SPR) sensor for the detection of MTC DNA [79]. In this approach, a graphene layer was deposited on an Au-SPR chip by drop-casting. Then, two different gold nanostructures, gold nanourchins (GNu) or gold nanorods (GNr), were used to label the single-stranded DNA (ssDNA) probe. This nanocomposite was then adsorbed onto the graphene layer, completing the biosensor assembly. In the presence of the target, complementary ssDNA (cssDNA) hybridisation resulted in the displacement of the probes from the graphene layer. This process was monitored by measuring the SPR signal. A comparison of GNu and GNr’s biorecognition signal revealed that the significant amplification of the plasmonic signal of GNu resulted in an LOD of ~24.5 fmol/L cssDNA. In contrast, the estimated signal of GNr reached only 8.2 pmol/L of cssDNA. Overall, this optical biosensor offered a simple, time-saving, and cost-effective design for the detection of MTC, while providing a low LOD and high specificity in DNA sensing, which is very promising for early screening of bacterial infections [79].

### 2.6. Other Zoonotic Agents

Campylobacteriosis is not considered a fatal disease in the EU. However, it does have an impact on morbidity. Campylobacteriosis is the second most commonly reported pathogen for foodborne outbreaks in the EU. Current data suggest that only 0.6% of human outbreaks are reported and investigated [13]. 

All *Campylobacter* species are non-spore-forming, microaerophilic, and sensitive to 3% or more NaCl concentrations. The cells are slender, spiral, and curved rods. However, some species are characterised by straighter rods, such as *C. showae* and *C. jejuni* [80]. The genus *Campylobacter* consists of 15 species and six subspecies. In Europe, 52.6% of confirmed campylobacteriosis cases are caused by *C. jejuni* (83.1%), followed by *C. coli* (10.8%), *C. lari* (0.1%), *C. fetus* (0.1%), and *C. upsaliensis* (0.1%) [13]. *C. jejuni* subsp. *jejuni,* a thermophilic organism, is often considered a commensal. It has been isolated from broiler chickens, particularly from the cecum, where body temperature is near 42 °C, and from the intestinal mucosa of mammals, where temperatures are lower [80].

The described outbreaks in Europe (94.5%) are associated with domestic infections [13], which supports the published results of Meldrum and Ribeiro (2003), who found no *Campylobacter* in RTE food [81]. Moreover, in this report, the percentage of contaminated RTE foods in 2019 is 6% compared to the 20% of non-RTE foods. The higher contamination of meat and meat products with this microorganism has been associated with domestic foodborne outbreaks, mainly due to cross-contamination of domestic environments [82]. Even low doses can lead to infection and illness [83]. To our knowledge, there is no published work by Portuguese teams on biosensors for *Campylobacter* spp. detection, which also represents an opportunity for innovative approaches.

Another important zoonotic agent is *Brucella* sp. It can be considered an ancient zoonosis and is still one of the most common bacterial zoonoses worldwide. Seven species of these Gram-negative, aerobic, facultative intracellular rods or coccobacilli infect terrestrial animals: *B. abortus*, *B. melitensis*, *B. suis*, *B. ovis*, *B. canis*, *B. neotomae*, and *B. microti* [84]. *Brucella melietensis* mainly cause human infection via infected tissues or secretions from infected animals, raw, improperly pasteurised, or unpasteurised dairy products. There are few foodborne outbreaks reported in the EU, only 17 between 2005 and 2019, related to cheese and raw milk as the main contaminated foods [13]. There is mandatory reporting of brucellosis in 26 member states of the EU. Confirmed cases are significantly decreasing in the population, except in Greece, with a reporting rate of 0.61/100,000, compared to the EU median of 0.06/100,000 [13]. Portugal also requires special attention, with 0.32 cases per 100,000 population, and unfortunately retains the status of “Not officially free” in 2019 [13]. 

The worldwide occurrence of this disease is underdiagnosed in many areas where it is endemic in livestock [85]. The gold standard test for it is the isolation of bacteria; however, sensitivity decreases by 20–30% for neurobrucellosis, with bone marrow cultures being more sensitive [86]. The most used screening tests are agglutination tests, such as Rose Bengal, antiglobulin, and Coomb’s test [85]. Nowadays, ELISA occupies a dominant position in laboratory settings due to its high sensitivity to detect specific IgM and IgG antibodies [87]. Another possible rapid test used is the fluorescence polarisation immunoassay (FPA), which offers high specificity (95%). The interpretation of all these rapid tests must be adapted to local epidemiological conditions, as specificity may decrease in endemic areas [88]. The only Portuguese work that developed a biosensor for *Brucella* sp. used a fluorescent immunoassay. Silva et al. (2004) established a competitive sandwich immunoreaction that allowed the quantification of anti-*Brucella sp.* antibodies in sheep serum samples, from 0.005 to 0.11 mg/mL [89].

A ubiquitous zoonotic pathogen of importance due to its antibiotic resistance is *Enterococcus* spp. *Enterococcus* can be classified as Gram-positive, spherical or ovoid cells, arranged in pairs or chains, non-spore-forming, facultatively anaerobic, and obligately fermentative [90]. The species, such as *Enterococcus faecalis* and *Enterococcus faecium*, have clinical importance, especially in hospitalised patients [90]. *E. faecalis* was ranked fourth (6.8%) in the list of most commonly reported microorganisms with antibiotic resistance. The most worrying increase was observed in the percentage of vancomycin-resistant isolates of *E. faecium* from 10.5% in 2015 to 18.3% in 2019 [65]. Thanks to genetic sequencing, new species have emerged exponentially in recent years that are not genetically virulent. Enterococci are ubiquitous in human faeces and persistent in the environment. Therefore, they are used as a water contamination indicator for human faeces and an indicator of hand hygiene [91]. Portuguese biosensors developed for the identification and quantification of enterococci are scarce. Lei et al. (2017) reported a highly sensitive micronuclear magnetic resonance (NMR) platform that detects 100 pmol/L of *E. faecalis*-derived DNA [92]. For this purpose, MNPs were functionalised with matched DNA as a probe to detect *E. faecalis* target DNA. Overall, this platform offers excellent advantages over available commercial NMR assay products by reducing sample consumption and hardware volume and weight [92].

**Figure 7:**
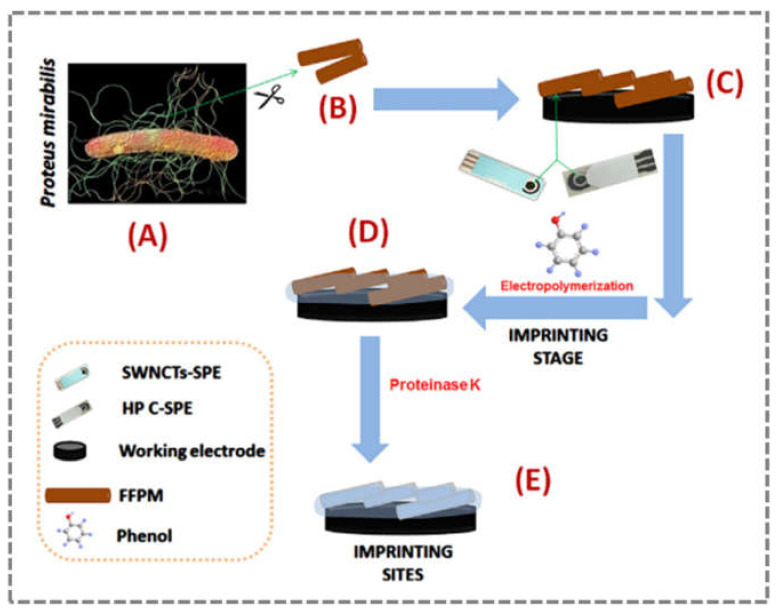
Scheme of MIP synthesis in the development of an impedance biosensor for rapid detection of foodborne pathogenic bacteria: *P. mirabilis* with flagellar filaments (FFPM) (**A**), which were removed from cells by shearing in a vortex with a glass bar and then passing repetitively through a syringe (**B**), and then FFPM were immobilised at the working area of SWCNTs-SPE/HP C-PE (**C**), followed by imprinting through electropolymerisation of phenol in acetate buffer (**D**), and binding sites formed after template removal by proteinase K (**E**) (reproduced with permission from Khan et al. (2017) [94], Copyright 2017, Elsevier B.V.).

Another zoonotic pathogen of particular importance in hospitalised patients is *Proteus mirabilis*. This bacterium is a Gram-negative motile rod, urease-positive, lactose-negative, and produces hydrogen sulfite [93]. Human infection by *P. mirabilis* can occur through swarming behaviour, adherence to and movement along surfaces, such as catheters, intravenous lines, and other medical devices. To date, only one biosensor for the detection of *P. mirabilis* has been developed in Portugal, by Khan et al. (2017) [94]. The team used a molecularly imprinted polymer (MIP) as artificial receptors to detect bacterial flagellar filaments (Figure 7). First, the bacterial flagellar filaments were adsorbed onto the carbon surface of the SPEs, followed by the formation of a polymeric phenolic network grown around the target. The final step consisted of the removal of the biomolecule from the polymeric network. After removal, complementary voids of the biomolecule formed, resulting in a molecularly imprinted polymer. The recognition process was followed by electrochemical techniques based on EIS and square-wave voltammetry (SWV), and flagellar filaments were detected at concentrations as low as 0.7 ng/mL by EIS and 0.9 ng/mL by SWV. The performance of these artificial receptors was an excellent result compared to natural antibodies. In addition, homemade paper-printed electrodes were also tested, which showed an LOD of 0.6 ng/mL by SWV, with the significant advantages of using paper as a support, i.e., the cost of such a portable, more environmentally friendly sensing device was significantly reduced [94].

### 2.7. Multiplex Biosensors

**Figure 8:**
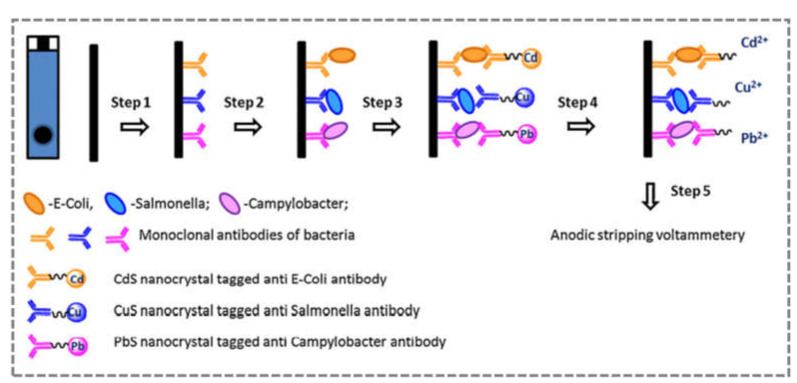
Scheme for multiplex detection of pathogens using nanocrystal antibody conjugates and MWCNT-PAH/SPE. Step 1 is the immobilisation of antibodies, followed by immunocapture (step 2), immunobinding of the conjugates (step 3), dissolution of metal ions from nanocrystals (step 4), and the SWASV analysis (step 5). (Reproduced with permission from Viswanathan et al. (2012) ([95], Copyright 2012, Elsevier B.V.).

Incorporating nanomaterials into the biosensor design affects its analytical performance and may open up the possibility of simultaneous multiplex detection of different pathogens. In this context, Viswanathan et al. (2012) fabricated an immunosensor by immobilising a mixture of anti-*E.coli*, anti-*Campylobacter*, and anti-*Salmonella* antibodies on the SPE surface modified with multi-walled carbon nanotubes and polyallylamine (MWCNT-PAH/SPE) (Figure 8) [95]. This sandwich immunoassay was performed with three antibodies conjugated with specific nanocrystals (*E. coli*–CdS, *Campylobacter*–PbS, and *Salmonella*–CuS), which allowed for different, non-overlapping stripping curves obtained by SWASV. In this work, linear responses were reported between 1 × 10^3^ and 5 × 10^5^ cells/mL, with an LOD of 800 cells/mL for *E. coli*, 400 cells/mL for *Salmonella*, and 400 cells/mL for *Campylobacter* [95].

In the work of Bessa Pereira et al. (2016), another multiplex platform was proposed in which the ability of scavenger receptor cysteine-rich (SRCR) functional groups to recognise and differentiate pathogen-associated molecular patterns of bacteria, fungi, or other microbes was investigated [96]. An antibody-free SPR-based assay was developed to increase the sensitivity of detection of extracellular proteins that bind to bacteria as a novel approach to overcome the sensitivity limitations of current Western blot methods. In this assay, proteins such as the N-terminal SRCR-containing portion of SSc5D (N-SSc5D), soluble Spα, and the extracellular domains of CD5 and CD6 were covalently bound to the sensor chip, which was previously functionalised with a self-assembling monolayer. Finally, SPR experiments showed that the receptors N-SSc5D and Spα could discriminate different bacteria (e.g., *L. monocytogenes* and *E. coli*) and different strains of *E. coli* [96].

An MR biochip was recently used to genetically analyse the 16S rRNA gene in the multiplex detection of five important mastitis-causing bacteria, including *E. coli*, *Klebsiella* sp., *S. aureus, Streptococcus uberis*, and *Streptococcus agalactiae* [29]. These MR biochips consisted of an array of 30 magnetic field sensors (spin valves) modified with a series of oligonucleotide probes representing the most common mastitis-causing bacteria. This was followed by amplification of the five other bacterial targets by asymmetric Polymerase Chain Reaction (PCR), which allowed successful detection of the targets without cross-reactivity and an LOD of 10^3^ cell/mL [29].

## 3. Conclusions and Future Perspectives

The detection of pathogens has always been a topic that encompasses the most conventional methods to innovative portable technologies. Especially for bacteria, new research has been proposed to overcome the tedious and time-consuming classical culture methods. The emergence of affordable and portable methods, such as biosensors and the incorporation of nanomaterials for improved analytical properties, are promising approaches.

In the specific context of Portuguese research, there is an obvious interest in the development of biosensors targeting *E. coli*, *Salmonella*, *L. monocytogenes*, and *S. aureus*. Given the relevance for improving quality control in the food industry and environmental monitoring, we are confident that progress will soon be made on other relevant pathogenic bacteria. In addition, the current pandemic caused by SARS-CoV-2 has highlighted the need for novel biosensors that enable cost-effective, rapid, and sensitive detection of pathogens that pose a threat to health. In parallel with global efforts, Portuguese researchers in the field of biosensors and point-of-use devices are driven by public health concerns and needs. Among them, there were only a limited number of studies that included multiplex analyses. 

Overall, there is still an open road ahead where simple, integrated, miniaturised systems can be part of routine diagnostics for hospital infections, food inspection, and veterinary/health surveillance. The move towards disposable sensors also brings new challenges, such as the need to adopt more environmentally friendly materials and processes that can be used in resource-limited environments. The ongoing innovation emanating from Portuguese researchers converges with EU R&I programmes aiming at improving the fight against zoonotic pathogens, with interesting future prospects.

## Figures and Tables

**Table 1:** Quantitative comparison between Portuguese (630 records) and worldwide research (68,680 records) on the distribution of publications in biosensors among the specified areas. Data obtained from webofknowledge.com cross-searching keywords: ALL FIELDS: (biosensor) and ADDRESS: (Portugal), and only keyword: ALL Fields: (Biosensor) in Web of Science [v.5.34]—Web of Science Core Collection Result Analysis (webofknowledge.com).

Number of Publications with Biosensor		
Area	Worldwide	Portuguese
Chemistry analytical	27,585	299
Electrochemistry	15,750	199
Nanoscience nanotechnology	12,328	93
Biotechnology applied microbiology	9096	77
Instrument instrumentation	7972	75
Biophysics	7806	93
Chemistry multidisciplinary	7161	63
Materials science multidisciplinary	6793	40
Physics applied	5792	43
Engineering, electrical and electronic	5774	48

## Data Availability

Not applicable.

## References

[1] Dincer C., Bruch R., Costa-Rama E., Fernández-Abedul M.T., Merkoçi A., Manz A., Urban G.A., Güder F. (2019). Disposable Sensors in Diagnostics, Food, and Environmental Monitoring. Adv. Mater..

[2] Clark L.C., Lyons C. (1962). Electrode systems for continuous monitoring in cardiovascular surgery. Ann. N. Y. Acad. Sci..

[3] Tothill I.E., Turner A.P.F., Caballero B. (2003). Biosensors. Encyclopedia of Food Sciences and Nutrition.

[4] Dargaville T.R., Farrugia B.L., Broadbent J.A., Pace S., Upton Z., Voelcker N.H. (2013). Sensors and imaging for wound healing: A review. Biosens. Bioelectron..

[5] Dang Y.T.H., Gangadoo S., Rajapaksha P., Truong V.K., Cozzolino D., Chapman J., Cifuentes A. (2021). Biosensors in food traceability and quality. Comprehensive Foodomics.

[6] Marko P.B., Lee S.C., Rice A., Gramling J.M., Fitzhenry T.M., McAlister J.S., Harper G., Moran A.L. (2004). Mislabelling of a depleted reef fish. Nature.

[7] Miller D., Jessel A., Mariani S. (2011). Seafood mislabelling: Comparisons of two Western European case studies assist in defining influencing factors, mechanisms and motives. Fish Fish..

[8] Mystery Fish: Seafood Fraud in Canada and How to Stop It. Oceana. http://www.oceana.ca/en/publications/reports/mystery-fish-seafood-fraud-canada-and-how-stop-it.

[9] Fish Substitution, 2015. European Commision Website. https://ec.europa.eu/food/food/agri-food-fraud/eu-coordinated-actions/coordinated-control-plans/fish-substitution-2015_en.

[10] Havelaar A.H., Kirk M.D., Torgerson P.R., Gibb H.J., Hald T., Lake R.J., Praet N., Bellinger D.C., de Silva N.R., Gargouri N. (2015). World Health Organization Global estimates and regional comparisons of the burden of foodborne disease in 2010. PLoS Med..

[11] Tavares A.P.M., Truta L.A., Moreira F.T.C., Carneiro L.P., Sales M.G.F. (2019). Self-powered and self-signalled autonomous electrochemical biosensor applied to cancinoembryonic antigen determination. Biosens. Bioelectron..

[12] Resende S., Frasco M., Sales M.G.F. (2020). A biomimetic photonic crystal sensor for label-free detection of urinary venous thromboembolism biomarker. Sens. Actuators B Chem..

[13] European Food Safety Authority, European Centre for Disease Prevention and Control (2021). The European Union one health 2019 zoonoses report. EFSA J..

[14] Ellermeier C.D., Slauch J.M., Dworkin M., Falkow S., Rosenberg E., Schleifer J.-H., Stanckebrandt E. (2006). The genus *Salmonella*. The Procaryotes—A Handbook on the Biology of Bacteria.

[15] Bailey S., Richardson L.J., Cox N.A., Cosby D.E., Juneja V.K., Sofos J.N. (2010). Chapter 7: *Salmonella*. Pathogens and Toxins in foods: Challenges and Interventions.

[16] Sant’Ana A.S., Franco B.D.G.M., Schaffner D.W. (2014). Risk of infection with *Salmonella* and *Listeria monocytogenes* due to consumption of ready-to-eat leafy vegetables in Brazil. Food Control.

[17] Freitas M., Viswanathan S., Nouws H.P., Oliveira M.B., Delerue-Matos C. (2014). Iron oxide/gold core/shell nanomagnetic probes and CdS biolabels for amplified electrochemical immunosensing of *Salmonella typhimurium*. Biosens. Bioelectron..

[18] Fernandes E., Martins V., Nóbrega C., Carvalho C., Cardoso F., Cardoso S., Dias J., Deng D., Kluskens L., Freitas P. (2014). A bacteriophage detection tool for viability assessment of *Salmonella* cells. Biosens. Bioelectron..

[19] Silva N.F., Magalhães J.M., Freire C., Delerue-Matos C. (2018). Electrochemical biosensors for *Salmonella*: State of the art and challenges in food safety assessment. Biosens. Bioelectron..

[20] Shen Y., Xu L., Li Y. (2021). Biosensors for rapid detection of *Salmonella* in food: A review. Compr. Rev. Food Sci. Food Saf..

[21] Serres M.H., Riley M., Dworkin M., Falkow S., Rosenberg E., Schleifer J.-H., Stanckebrandt E. (2006). Genomics and metabolism in *Escherichia coli*. The Procaryotes—A Handbook on the Biology of Bacteria.

[22] Buchanan R., Edelson S. (1999). Effect of pH-dependent, stationary phase acid resistance on the thermal tolerance of *Escherichia coli* O157:H7. Food Microbiol..

[23] Johnson J.R., Oswald E., O’Bryan T.T., Kuskowski M.A., Spanjaard L. (2002). Phylogenetic distribution of virulence-associated genes among Escherichia coli isolates associated with neonatal bacterial meningitis in The Netherlands. J. Infect. Dis..

[24] Begue R.E., Mehta D.I., Blecker U. (1998). *Escherichia coli* and the Hemolytic-uremic syndrome. South. Med. J..

[25] Bacon R.T., Ransom J.R., Sofos J.N., Kendall P.A., Belk K.E., Smith G.C. (2003). Thermal inactivation of susceptible and multiantimicrobial-resistant *Salmonella* strains grown in the absence or presence of glucose. Appl. Environ. Microbiol..

[26] Buchanan R.L., Doyle M.P. (1997). Foodborne disease significance of *Escherichia coli* O157:H7 and other enterohemorrhagic *E. coli*. Food Technol..

[27] Guion C.E., Ochoa T.J., Walker C.M., Barletta F., Cleary T.G. (2008). Detection of diarrheagenic *Escherichia coli* by Use of melting-curve analysis and real-time multiplex PCR. J. Clin. Microbiol..

[28] Nataro J.P., Martinez J., Woodford N., Johnson A. (2003). Diagnosis and investigation of diarrheagenic *Escherichia coli*. Mol. Bacteriol..

[29] Viveiros S., Rodrigues M., Albuquerque D., Martins S.A.M., Cardoso S., Martins V.C. (2020). Multiple bacteria identification in the point-of-care: An old method serving a new approach. Sensors.

[30] Barreiros dos Santos M., Agusil J., Prieto-Simon B., Sporer C., Teixeira V., Samitier J. (2013). Highly sensitive detection of pathogen *Escherichia coli* O157:H7 by electrochemical impedance spectroscopy. Biosens. Bioelectron..

[31] Couto R.A.S., Chen L., Kuss S., Compton R.G. (2018). Detection of *Escherichia coli* bacteria by impact electrochemistry. Analyst.

[32] Kuss S., Couto R.A.D.S., Evans R., Lavender H., Tang C.C., Compton R.G. (2019). Versatile electrochemical sensing platform for bacteria. Anal. Chem..

[33] Queirós R.B., De-Los-Santos-Álvarez N., Noronha J.P., Sales M.G.F. (2013). A label-free DNA aptamer-based impedance biosensor for the detection of *E. coli* outer membrane proteins. Sens. Actuators B Chem..

[34] Barreiros dos Santos M., Azevedo S., Agusil J., Prieto-Simon B., Sporer C., Torrents E., Juárez A., Teixeira V., Samitier J. (2015). Label-free ITO-based immunosensor for the detection of very low concentrations of pathogenic bacteria. Bioelectrochemistry.

[35] Martins S.A.M., Prazeres D.M.F., Fonseca L.P., Monteiro G.A. (2009). Quantitation of non-amplified genomic DNA by bead-based hybridization and template mediated extension coupled to alkaline phosphatase signal amplification. Biotechnol. Lett..

[36] Mouffouk F., da Costa A.R., Martins J., Zourob M., Abu-Salah K.M., Alrokayan S. (2011). Development of a highly sensitive bacteria detection assay using fluorescent pH-responsive polymeric micelles. Biosens. Bioelectron..

[37] Queirós R., Gouveia C., Fernandes J.R.A., Jorge P. (2014). Evanescent wave DNA-aptamer biosensor based on long period gratings for the specific recognition of E. coli outer membrane proteins. Biosens. Bioelectron..

[38] Kokkinis G., Cardoso S.F., Cardoso F.A., Giouroudi I. (2014). Microfluidics for the rapid detection of pathogens using giant magnetoresistance sensors. IEEE Trans. Magn..

[39] Bastos A.R., Vicente C.M.S., Oliveira-Silva R., Silva N.J.O., Tacão M., Da Costa J.P., Lima M.J.S., André P.S., Ferreira R.A.S. (2018). Integrated optical mach-zehnder interferometer based on organic-inorganic hybrids for photonics-on-a-chip biosensing applications. Sensors.

[40] Rocourt J., Buchrieser C., Ryser E.T., Marth A.H. (2007). Chapter 1 The Genus *Listeria* and *Listeria monocytogenes*: Phylogenetic Position, Taxonomy, and Identification. Literia, Listeriosis, and Food Safety.

[41] Khelef N., Lecuit M., Buchrieser C., Cabanes D., Dussurget O., Cossart P., Dworkin M., Falkow S., Rosenberg E., Schleifer J.-H., Stanckebrandt E. (2006). *Listeria monocytogenes* and the Genus Listeria. The Procaryotes—A Handbook on the Biology of Bacteria.

[42] Ben Embarek P.K. (1994). Presence, detection and growth of *Listeria monocytogenes* in seafoods: A review. Int. J. Food Microbiol..

[43] Painter J., Slutsker L., Ryser E.T., Marth E.H. (2007). Chapter 4: Listeriosis in humans. Listeria, Listeriosis, and Food Safety.

[44] Hitchins A.D., Whiting R.C. (2001). Food-borne *Listeria monocytogenes* risk assessment. Food Addit. Contam..

[45] Tompkin R.B. (2002). Control of *Listeria monocytogenes* in the food-processing environment. J. Food Prot..

[46] McLauchlin J. (1996). The relationship between Listeria and listeriosis. Food Control.

[47] Braga V., Vázquez S., Vico V., Pastorino V., Mota M.I., Legnani M., Schelotto F., Lancibidad G., Varela G. (2017). Prevalence and serotype distribution of *Listeria monocytogenes* isolated from foods in Montevideo-Uruguay. Braz. J. Microbiol..

[48] Schlech W.F., Lavigne P.M., Bortolussi R.A., Allen A.C., Haldane E.V., Wort A.J., Hightower A.W., Johnson S.E., King S.H., Nicholls E.S. (1983). Epidemic Listeriosis—Evidence for transmission by food. N. Engl. J. Med..

[49] Federighi M., Declerq E., Jugiau F., Cappelier J.-M. (2002). Environmental and physico-chemical factors induce VBNC state in *Listeria monocytogenes*. Veter. Res..

[50] Bille J. (1990). Epidemiology of Human Listeriosis in Europe, with Special Reference to the Swiss Outbreak.

[51] McIntyre L., Wilcott L., Naus M. (2015). Listeriosis outbreaks in British Columbia, Canada, caused by soft ripened cheese contaminated from environmental sources. BioMed Res. Int..

[52] Gilbert R.J., McLauchlin J., Velani S.K. (1993). The contamination of paté *byListeria monocytogenesin* England and Wales in 1989 and 1990. Epidemiol. Infect..

[53] Jacquet C., Catimel B., Brosch R., Buchrieser C., Dehaumont P., Goulet V., Lepoutre A., Veit P., Rocourt J. (1995). Investigations related to the epidemic strain involved in the French listeriosis outbreak in 1992. Appl. Environ. Microbiol..

[54] Dalton C.B., Austin C.C., Sobel J., Hayes P.S., Bibb W.F., Graves L.M., Swaminathan B., Proctor M.E., Griffin P.M. (1997). An Outbreak of gastroenteritis and fever due to *Listeria monocytogenesin* milk. N. Engl. J. Med..

[55] Salamina G., Donne E.D., Niccolini A., Poda G., Cesaroni D., Bucci M., Fini R., Maldini M., Schuchat A., Swaminathan B. (1996). A foodborne outbreak of gastroenteritis involvingListeria monocytogenes. Epidemiol. Infect..

[56] Farber J., Daley E., Mackie M., Limerick B. (2000). A small outbreak of listeriosis potentially linked to the consumption of imitation crab meat. Lett. Appl. Microbiol..

[57] Soni D.K., Ahmad R., Dubey S.K. (2018). Biosensor for the detection of *Listeria monocytogenes*: Emerging trends. Crit. Rev. Microbiol..

[58] Silva N.F.D., Neves M.M., Magalhães J.M., Freire C., Delerue-Matos C. (2020). Electrochemical immunosensor towards invasion-associated protein p60: An alternative strategy for *Listeria monocytogenes* screening in food. Talanta.

[59] Seo K.S., Bohach G.A., Juneja V.K., Sofos J.N. (2010). Chapter 8: Staphylococcal food poisoning. Pathogens and Toxins in Foods: Challenges and Interventions.

[60] Ray B., Bhunia A., Ray B., Bhunia A. (2008). Foodborne intoxication. Fundamental Food Microbiology.

[61] Gotz F., Bannerman T., Schleifer J.-H., Dworkin M., Falkow S., Rosenberg E., Schleifer J.-H., Stanckebrandt E. (2006). The genus staphylococcus and macrococcus. The Procaryotes—A Handbook on the Biology of Bacteria.

[62] Kloos W.E. (1980). Natural populations of the genus staphylococcus. Annu. Rev. Microbiol..

[63] Lowy F.D. (1998). *Staphylococcus aureus* infections. N. Engl. J. Med..

[64] Ryu S., Song P.I., Seo C.H., Cheong H., Park Y. (2014). Colonization and infection of the skin by *S. aureus*: Immune system evasion and the response to cationic antimicrobial peptides. Int. J. Mol. Sci..

[65] European Centre for Disease Prevention and Control Country Summaries-Antimicrobial Resistance in the EU/EEA (EARS-Net) Annual Epidemiological Report for 2019. https://www.ecdc.europa.eu/en/publications-data/surveillance-antimicrobial-resistance-europe-2019.

[66] Gautam S., Kim T., Shoda T., Sen S., Deep D., Luthra R., Ferreira M.T., Pinho M.G., Spiegel D.A. (2015). An activity-based probe for studying crosslinking in live bacteria. Angew. Chem. Int. Ed..

[67] Duarte C.M., Carneiro C., Cardoso S., De Freitas S.C., Bexiga R. (2016). Semi-quantitative method for Staphylococci magnetic detection in raw milk. J. Dairy Res..

[68] Behr M.A., Gagneux S. (2011). The rise and fall of the *Mycobacterium tuberculosis* complex. Genetics and Evolution of Infectious Disease.

[69] Velayati A.A., Farnia P. (2017). Atlas of *Mycobacterium tuberculosis*. Atlas of Myobacterium Tuberculosis.

[70] World Health Organization (2020). Global Tuberculosis Report.

[71] Houben R.M.G.J., Dodd P.J. (2016). The global burden of latent tuberculosis infection: A re-estimation using mathematical modelling. PLoS Med..

[72] Barroso T.G., Martins R., Fernandes E., Cardoso S., Rivas J., Freitas P. (2018). Detection of BCG bacteria using a magnetoresistive biosensor: A step towards a fully electronic platform for tuberculosis point-of-care detection. Biosens. Bioelectron..

[73] Garberi J.E., Labrador J., Garberi F., Peneipil J., Garberi M., Scigliano L., Troncoso A. (2012). Rapid and biosecure diagnostic test for tuberculosis. Cell Biophys..

[74] Barroso T.R., Martins V.C., Cardoso F., Cardoso S., Pedrosa J., Correia-Neves M., Rivas J., De Freitas S.C. (2015). Detecting antibody-labeled BCG MNPs using a magnetoresistive biosensor and magnetic labeling technique. J. Nano Res..

[75] Bernacka-Wojcik I., Senadeera R., Wojcik P.J., Silva L.B., Doria G., Baptista P., Águas H., Fortunato E., Martins R. (2010). Inkjet printed and “doctor blade” TiO_2_ photodetectors for DNA biosensors. Biosens. Bioelectron..

[76] Baptista P.V., Koziol-Montewka M., Paluch-Oles J., Doria G., Franco R. (2006). Gold-nanoparticle-probe–based assay for rapid and direct detection of *Mycobacterium tuberculosis* DNA in clinical samples. Clin. Chem..

[77] Doria G., Franco R., Baptista P. (2007). Nanodiagnostics: Fast colorimetric method for single nucleotide polymorphism/mutation detection. IET Nanobiotechnol..

[78] Silva L.B., Veigas B., Doria G., Costa P., Inácio J., Martins R., Fortunato E., Baptista P.V. (2011). Portable optoelectronic biosensing platform for identification of mycobacteria from the Mycobacterium tuberculosis complex. Biosens. Bioelectron..

[79] Prabowo B.A., Purwidyantri A., Liu B., Lai H.-C., Liu K.-C. (2021). Gold nanoparticle-assisted plasmonic enhancement for DNA detection on a graphene-based portable surface plasmon resonance sensor. Nanotechnology.

[80] Wagenaar J.A., Van Bergen M.A., Mueller M.A., Wassenaar T.M., Carlton R.M. (2005). Phage therapy reduces *Campylobacter jejuni* colonization in broilers. Veter. Microbiol..

[81] Meldrum R.J., Ribeiro C.D. (2003). Campylobacter in ready-to-eat foods: The result of a 15-month survey. J. Food Prot..

[82] Verhoeff-Bakkenes L., Beumer R.R., De Jonge R., Van Leusden F.M., De Jong A.E.I. (2008). Quantification of *Campylobacter jejuni* cross-contamination via hands, cutlery, and cutting board during preparation of a chicken fruit salad. J. Food Prot..

[83] Black R.E., Levine M.M., Clements M.L., Hughes T.P., Blaser M.J. (1988). Experimental *Campylobacter jejuni* infection in humans. J. Infect. Dis..

[84] Khurana S.K., Sehrawat A., Tiwari R., Prasad M., Gulati B., Shabbir M.Z., Chhabra R., Karthik K., Patel S.K., Pathak M. (2021). Bovine brucellosis—A comprehensive review. Veter. Q..

[85] Franco M.P., Mulder M., Gilman R.H., Smits H.L. (2007). Human brucellosis. Lancet Infect. Dis..

[86] De Glanville W.A., Conde-Álvarez R., Moriyon I., Njeru J., Díaz R., Cook E., Morin M., Bronsvoort B.M.D.C., Thomas L., Kariuki S. (2017). Poor performance of the rapid test for human brucellosis in health facilities in Kenya. PLoS Negl. Trop. Dis..

[87] Mantecón M.Á., Gutiérrez P., Zarzosa M.D.P., Dueñas A.I., Solera J., Fernández-Lago L., Vizcaino N., Almaraz A., Bratos M.A., Torres A.R. (2006). Utility of an immunocapture-agglutination test and an enzyme-linked immunosorbent assay test against cytosolic proteins from *Brucella melitensis* B115 in the diagnosis and follow-up of human acute brucellosis. Diagn. Microbiol. Infect. Dis..

[88] Ariza J., Pellicer T., Pallares R., Foz A., Gudiol F. (1992). Specific antibody profile in human brucellosis. Clin. Infect. Dis..

[89] Silva M., Cruz H., Rossetti O., Arese A., Oliva A. (2004). Development of an optical immunosensor based on the fluorescence of Cyanine-5 for veterinarian diagnostics. Biotechnol. Lett..

[90] Gilmore M., Clewell D., Ike Y., Shankar N. (2014). Enterococci: From Commensals to Leading Causes of Drug Resistant Infection [Internet].

[91] European Comission (2015). Commission Directive (EU) 2015/1787 of 6 October 2015 Amending Annexes II and III to Council Directive 98/83/EC on the Quality of Water Intended for Human Consumption.

[92] Lei K.-M., Heidari H., Mak P.-I., Law M.-K., Maloberti F., Martins R.P. (2016). A handheld high-sensitivity micro-NMR CMOS platform with B-field stabilization for multi-type biological/chemical assays. IEEE J. Solid State Circuits.

[93] Schaffer J.N., Pearson M.M. (2015). Proteus mirabilis and urinary tract infections. Microbiol. Spectr..

[94] Khan M.A.R., Cardoso A.R.A., Sales M.G.F., Merino S., Tomás J.M., Rius F.X., Riu J. (2017). Artificial receptors for the electrochemical detection of bacterial flagellar filaments from *Proteus mirabilis*. Sens. Actuators B Chem..

[95] Viswanathan S., Rani C., Ho A. (2012). Electrochemical immunosensor for multiplexed detection of food-borne pathogens using nanocrystal bioconjugates and MWCNT screen-printed electrode. Talanta.

[96] Pereira C.B., Bocková M., Santos R.F., Santos A.M., De Araújo M.M., Oliveira L., Homola J., Carmo A.M. (2016). The scavenger receptor SSc5D physically interacts with bacteria through the SRCR-containing N-terminal domain. Front. Immunol..

